# Impact of Exercise on Physiological, Biochemical, and Analytical Parameters in Patients with Heart Failure with Reduced Ejection Fraction

**DOI:** 10.3390/medicina60122017

**Published:** 2024-12-06

**Authors:** Francisco Epelde

**Affiliations:** Medicine Department, Parc Taulí Hospital Universitari, Institut d’Investigació i Innovació Parc Taulí I3PT, 08208 Sabadell, Spain; fepelde@gmail.com

**Keywords:** heart failure, exercise, cardiac rehabilitation

## Abstract

Heart failure with reduced ejection fraction (HFrEF) is a condition marked by diminished cardiac output and impaired oxygen delivery to tissues. Exercise, once avoided in HFrEF patients due to safety concerns, is now recognized as an important therapeutic intervention. Structured exercise improves various physiological, biochemical, and analytical parameters, including cardiac output, endothelial function, skeletal muscle performance, and autonomic regulation. Biochemically, exercise induces favorable changes in inflammatory markers, lipid profiles, glucose metabolism, and renal function. This paper reviews these changes, highlighting how exercise can be safely incorporated into HFrEF management. Further research is needed to tailor exercise interventions for individual patients to optimize outcomes.

## 1. Introduction

Heart failure with reduced ejection fraction (HFrEF) is a major global public health challenge, affecting millions of individuals worldwide and contributing significantly to healthcare costs. HFrEF is clinically defined by a left ventricular ejection fraction (LVEF) of less than 40%, which reflects the heart’s diminished ability to pump blood efficiently to meet the body’s metabolic demands [1]. This condition is often the result of myocardial injury, such as ischemic heart disease, which weakens the heart muscle. It leads to significant impairments in cardiac output and, consequently, the systemic circulation. In addition to the hemodynamic compromise, HFrEF is characterized by neurohormonal activation, chronic inflammation, and alterations in autonomic regulation, all of which contribute to the progression of the disease [2].

Patients with HFrEF commonly present with a wide range of symptoms, including fatigue, dyspnea, and exercise intolerance, which severely impair their quality of life. These symptoms are linked to the heart’s inability to adequately supply oxygenated blood during periods of increased demand, such as physical exertion [3]. As the disease advances, patients may also experience fluid retention, resulting in pulmonary and peripheral edema. The combination of these factors leads to increased hospitalizations, reduced functional independence, and heightened mortality rates. Globally, heart failure is a leading cause of hospitalization in individuals over the age of 65, and despite advances in pharmacological therapies, the prognosis for HFrEF remains poor, with a five-year mortality rate exceeding 50% [4].

Historically, the management of HFrEF has revolved around pharmacological therapies that aim to modulate the renin–angiotensin–aldosterone system (RAAS) and the sympathetic nervous system (SNS), both of which play central roles in the pathophysiology of heart failure. Medications such as angiotensin-converting enzyme inhibitors (ACEIs), beta-blockers, mineralocorticoid receptor antagonists, and, more recently, angiotensin receptor–neprilysin inhibitors (ARNIs) have been the cornerstone of treatment, significantly improving survival, reducing hospital admissions, and delaying disease progression. SGLT2 inhibitors (SGLT2is) have emerged as a cornerstone in the treatment of heart failure with reduced ejection fraction (HFrEF), significantly altering the management landscape for this condition. These medications, initially developed for diabetes management, work by inhibiting the sodium-glucose cotransporter 2 in the kidneys, leading to enhanced glycemic control and diuresis. This diuretic effect helps reduce fluid overload, a common issue in heart failure patients, thereby alleviating symptoms such as dyspnea and edema.

Moreover, SGLT2is have been shown to provide substantial cardiovascular benefits, including reduced hospitalizations due to heart failure and improved overall survival rates. Clinical trials have demonstrated that SGLT2is not only improve heart failure symptoms but also address underlying pathophysiological mechanisms, such as metabolic dysfunction and inflammation, contributing to the progression of heart failure.

Their ability to modulate renal function and promote favorable cardiac remodeling further solidifies their role in heart failure management. As ongoing research continues to uncover additional therapeutic potentials, SGLT2 inhibitors are becoming increasingly recognized as essential components of a comprehensive treatment strategy for HFrEF, offering hope for improved quality of life and outcomes in affected patients [5]. Despite these pharmacologic advancements, a large number of HFrEF patients continue to experience persistent symptoms, and additional strategies are needed to optimize patient outcomes.

In recent years, the role of non-pharmacological interventions, particularly exercise training, has gained increasing attention as an adjunctive therapy for managing HFrEF. Exercise intolerance is a hallmark feature of heart failure, driven by a combination of central (cardiac) and peripheral (muscular and vascular) factors [6]. For decades, there was hesitancy regarding the recommendation of exercise for HFrEF patients, largely due to concerns that physical exertion could exacerbate cardiac dysfunction and lead to adverse events. However, accumulating evidence from clinical trials and observational studies has since established the safety, efficacy, and beneficial effects of structured exercise programs in this population [7].

Exercise training in HFrEF has been shown to improve multiple physiological parameters, including increased peak oxygen uptake (VO_2peak_), enhanced endothelial function, and improved skeletal muscle metabolism. Furthermore, exercise modifies several biochemical markers of disease progression, such as inflammatory cytokines, oxidative stress, and neurohormonal activation. These adaptations not only translate to improved exercise capacity and symptom relief but also positively impact long-term clinical outcomes, including reduced hospitalization and mortality rates [8].

Moreover, exercise has been found to influence key analytical parameters in HFrEF patients, such as biomarkers of cardiac stress (e.g., B-type natriuretic peptide or BNP), which are commonly used to assess disease severity and guide treatment decisions [9]. Changes in these biomarkers following regular exercise may reflect improvements in ventricular function, reduced myocardial stress, and enhanced overall cardiovascular health.

Given the complex and multifactorial nature of HFrEF, the integration of exercise into a comprehensive management plan is essential for addressing the broad spectrum of pathophysiological changes that occur in this condition. This review aims to explore the effects of exercise on patients with HFrEF, focusing on its ability to modify cardiovascular, metabolic, biochemical, and analytical parameters. The evidence supporting the safety and efficacy of exercise interventions will be critically examined, and the underlying mechanisms through which exercise exerts its beneficial effects will be discussed. In doing so, this review will provide insight into how non-pharmacological strategies, particularly exercise, can complement traditional medical therapies to improve the prognosis and quality of life in patients with HFrEF.

## 2. Pathophysiology of HFrEF and Exercise Intolerance

Heart failure with reduced ejection fraction (HFrEF) is a complex and multifaceted syndrome, rooted in a combination of hemodynamic disturbances, neurohormonal dysregulation, and peripheral organ dysfunction. These interrelated processes not only drive the progression of heart failure but also contribute to the hallmark symptom of exercise intolerance, a major determinant of poor quality of life in affected individuals [10]. The defining feature of HFrEF is the impaired systolic function of the left ventricle, typically measured by a left ventricular ejection fraction (LVEF) of less than 40%. This impaired contractility leads to an inability of the heart to adequately pump blood, particularly during periods of increased demand, such as exercise. Consequently, the reduced cardiac output limits oxygen delivery to peripheral tissues, contributing to exercise intolerance and fatigue, which are hallmark symptoms of the syndrome [11].

At the core of HFrEF is a persistent imbalance between the heart’s pumping capacity and the body’s metabolic demands. In response to a reduction in cardiac output, the body initiates several compensatory mechanisms aimed at preserving perfusion to vital organs. Two primary systems are activated: the renin–angiotensin–aldosterone system (RAAS) and the sympathetic nervous system (SNS) [12]. These systems, although beneficial in the short term, have deleterious effects when chronically activated. The RAAS is responsible for fluid retention, sodium reabsorption, and vasoconstriction, all of which increase blood volume and, consequently, cardiac preload and afterload. Meanwhile, the activation of the SNS increases heart rate and contractility while also inducing peripheral vasoconstriction to maintain systemic blood pressure in the face of declining cardiac function [13].

Although these mechanisms temporarily help to maintain cardiac output and tissue perfusion, their chronic activation contributes to a vicious cycle of worsening heart failure. Elevated levels of circulating angiotensin II and aldosterone lead to progressive vasoconstriction, increased afterload, and sodium and fluid retention. These effects exacerbate the workload on the failing heart, leading to further myocardial strain and progressive deterioration in cardiac function. The heart, in an effort to compensate for this increased workload, undergoes structural adaptations, including left ventricular dilation, increased wall stress, and fibrosis—a process known as adverse cardiac remodeling [14]. Over time, this remodeling worsens systolic function and leads to further reductions in ejection fraction, perpetuating a downward spiral of heart failure progression.

The process of adverse remodeling is driven not only by mechanical stress but also by neurohormonal activation and inflammation. Chronic SNS activation leads to increased levels of circulating catecholamines, such as norepinephrine, which promote hypertrophy of the cardiac muscle, apoptosis (cell death), and fibrosis [15]. Similarly, RAAS activation leads to elevated levels of angiotensin II, which triggers inflammatory responses within the myocardium, further contributing to fibrosis and the stiffening of the heart walls. This fibrosis reduces the heart’s ability to contract and relax efficiently, impairing both systolic and diastolic function. These pathological changes are not confined to the heart alone but also have systemic effects, influencing other organs such as the kidneys, lungs, and skeletal muscles [16].

One of the most profound consequences of HFrEF is the development of exercise intolerance, which is a cardinal feature of the syndrome. The underlying mechanisms of exercise intolerance in HFrEF are complex and multifactorial. While reduced cardiac output is the primary factor, peripheral factors play a significant role. In addition to decreased oxygen delivery to skeletal muscles, patients with HFrEF exhibit impaired skeletal muscle metabolism, abnormal vasodilation, and a shift in muscle fiber type composition [17]. The muscle fibers of HFrEF patients often shift from oxidative (type I) fibers, which are fatigue-resistant, to glycolytic (type II) fibers, which fatigue more rapidly. This shift reduces the muscle’s efficiency during exercise, further compounding exercise intolerance [18].

Peripheral endothelial dysfunction also contributes to exercise limitations in HFrEF patients. Normally, exercise leads to the release of nitric oxide from endothelial cells, promoting vasodilation and increasing blood flow to active muscles. However, in HFrEF, endothelial dysfunction blunts this response, leading to inadequate blood flow and oxygen delivery to the muscles during physical exertion. Moreover, oxidative stress and chronic inflammation, both of which are upregulated in HFrEF, further impair endothelial function and exacerbate skeletal muscle abnormalities. The end result is a combination of central (cardiac) and peripheral (vascular and muscular) limitations that severely reduce exercise capacity in these patients [19].

In addition to peripheral vascular and muscular abnormalities, neurohormonal alterations play a significant role in the pathophysiology of exercise intolerance. The chronic elevation of circulating catecholamines and the overstimulation of the SNS contribute to exaggerated ventilatory responses to exercise, known as exercise hyperpnea [20]. This increased respiratory effort, coupled with the sensation of dyspnea, significantly limits physical performance and discourages physical activity, leading to a cycle of deconditioning that further worsens exercise capacity. Skeletal muscle dysfunction is a significant and often overlooked aspect of heart failure, particularly in patients with reduced ejection fraction. This condition manifests as a reduction in muscle mass and strength, which can lead to severe impairment in physical performance and exercise intolerance. In heart failure, various metabolic changes occur, including a decrease in the availability of amino acids, which are critical for muscle protein synthesis. This deficiency can trigger the mobilization of protein reserves from skeletal muscle, exacerbating muscle wasting and further compromising the functional capacity of these patients.

The interplay between heart failure and skeletal muscle dysfunction creates a vicious cycle; as patients experience fatigue and weakness, their ability to engage in physical activity diminishes, leading to further deconditioning. This decline in muscle function not only impacts daily activities, such as climbing stairs or walking short distances, but also affects overall quality of life. Additionally, skeletal muscle dysfunction is associated with worse clinical outcomes, including increased morbidity and mortality.

Emerging evidence suggests that targeted interventions, particularly exercise training, can significantly improve skeletal muscle function in heart failure patients. Exercise has been shown to enhance muscle strength, increase endurance, and improve metabolic flexibility. Furthermore, it can help mitigate the effects of muscle wasting, thereby improving overall physical performance and quality of life. Addressing skeletal muscle health through comprehensive rehabilitation programs is essential for enhancing functional capacity, promoting independence, and ultimately improving outcomes for individuals living with heart failure. By recognizing and treating skeletal muscle dysfunction, healthcare providers can offer more effective, holistic care to heart failure patients [21].

## 3. Exercise Intolerance in HFrEF

Exercise intolerance is a cardinal feature of heart failure with reduced ejection fraction (HFrEF) and is a major contributor to the poor quality of life in these patients. The inability to perform even moderate physical activity is one of the most common complaints among individuals with HFrEF and is a critical predictor of adverse outcomes. This exercise intolerance results from a combination of central and peripheral mechanisms that interact to limit the cardiovascular system’s ability to respond adequately to physical exertion [22].

### 3.1. Central Mechanisms of Exercise Intolerance

At the core of exercise intolerance in HFrEF are the central hemodynamic abnormalities associated with impaired cardiac function. The primary central factors contributing to exercise intolerance include reduced cardiac output during exercise, impaired ventricular filling due to elevated left ventricular end-diastolic pressure, and an inability to appropriately increase heart rate and stroke volume during physical exertion. In healthy individuals, exercise triggers an increase in cardiac output driven by both increased stroke volume and heart rate, which facilitates the delivery of oxygen and nutrients to working muscles. However, in HFrEF, the heart’s ability to augment stroke volume in response to exercise is significantly impaired due to systolic dysfunction and increased afterload, resulting from elevated systemic vascular resistance [23].

Additionally, elevated left ventricular end-diastolic pressure and impaired ventricular relaxation contribute to diastolic dysfunction. This diastolic dysfunction limits ventricular filling during exercise, reducing preload and impairing the Frank–Starling mechanism, which normally enhances stroke volume as the heart fills with more blood. Moreover, the inability to sufficiently increase heart rate in response to exercise, termed chronotropic incompetence, further limits the increase in cardiac output necessary to meet the metabolic demands of exercising muscles [24]. As a result, reduced blood flow to peripheral tissues limits oxygen and nutrient delivery, constraining exercise capacity.

Another significant central factor is the redistribution of blood flow during exercise. In healthy individuals, blood flow is preferentially redirected toward skeletal muscles and away from non-essential organs during physical exertion. However, in HFrEF, this redistribution is blunted due to impaired vascular regulation and persistent neurohormonal activation, further limiting the supply of oxygen to exercising muscles [25]. This mismatch between oxygen demand and supply in peripheral tissues leads to early fatigue, dyspnea, and a profound sense of physical exhaustion, all of which contribute to exercise intolerance in HFrEF patients.

### 3.2. Peripheral Mechanisms of Exercise Intolerance

Although central hemodynamic factors play a pivotal role in limiting exercise capacity, peripheral abnormalities in skeletal muscle, vascular function, and metabolic processes are equally important contributors to exercise intolerance in HFrEF. A key peripheral factor is skeletal muscle dysfunction, which is characterized by reduced capillary density, mitochondrial dysfunction, and muscle fiber-type changes [26].

#### 3.2.1. Skeletal Muscle Dysfunction

Patients with HFrEF commonly exhibit skeletal muscle abnormalities, including a shift in muscle fiber composition [27]. There is a reduction in oxidative (type I) muscle fibers, which are responsible for sustained aerobic activity, and an increase in glycolytic (type II) fibers, which rely more on anaerobic metabolism and fatigue more quickly. This fiber-type shift leads to a reduced oxidative capacity and an increased reliance on anaerobic pathways, which generate less energy and produce lactate, leading to early-onset muscle fatigue. This shift in muscle fiber composition is exacerbated by muscle disuse and deconditioning as patients with HFrEF often reduce their physical activity due to their symptoms. 

#### 3.2.2. Mitochondrial Dysfunction

Mitochondrial dysfunction is another critical factor contributing to exercise intolerance in HFrEF. Mitochondria are essential for producing adenosine triphosphate (ATP), the primary energy source for muscle contraction during aerobic exercise, through oxidative phosphorylation. In patients with HFrEF, mitochondrial biogenesis is impaired, resulting in a reduction in both mitochondrial content and function within skeletal muscles. This reduction in mitochondrial density and oxidative capacity forces muscles to rely on less efficient anaerobic pathways, leading to increased lactate production and early fatigue. The inability of skeletal muscles to efficiently use oxygen for ATP production limits endurance and further exacerbates exercise intolerance [28].

#### 3.2.3. Endothelial Dysfunction

Another key peripheral factor is endothelial dysfunction, which plays a significant role in regulating blood flow and vascular tone during exercise. Under normal conditions, endothelial cells release nitric oxide (NO), a potent vasodilator, in response to exercise. This NO-mediated vasodilation increases blood flow to active muscles, allowing for greater oxygen delivery. However, in HFrEF, endothelial dysfunction results in decreased NO production and increased oxidative stress, impairing the ability of blood vessels to dilate properly. This leads to reduced blood flow to skeletal muscles during exercise, further limiting oxygen delivery and contributing to early muscle fatigue. Additionally, increased levels of circulating inflammatory markers, such as tumor necrosis factor-alpha (TNF-α), contribute to endothelial damage and exacerbate vascular dysfunction in HFrEF [29,30].

#### 3.2.4. Muscle Atrophy and Sarcopenia

Skeletal muscle atrophy, or sarcopenia, is commonly observed in patients with HFrEF and is another important contributor to exercise intolerance. Sarcopenia is characterized by a loss of muscle mass and strength, which reduces the muscle’s ability to generate force and sustain activity. This muscle wasting is driven by a combination of factors, including reduced anabolic signaling, increased protein degradation, and chronic systemic inflammation [22]. In HFrEF, reduced levels of insulin-like growth factor-1 (IGF-1) and other anabolic hormones lead to impaired muscle protein synthesis, while increased levels of pro-inflammatory cytokines promote muscle protein breakdown. The resulting reduction in muscle mass decreases the strength and endurance of the muscles, further contributing to the overall reduction in exercise capacity [31,32].

### 3.3. The Vicious Cycle of Reduced Activity and Deconditioning

The interplay between central and peripheral mechanisms creates a vicious cycle in HFrEF patients, where reduced physical activity leads to further deconditioning, worsening muscle function, and declining cardiovascular health. As patients with HFrEF experience symptoms such as dyspnea and fatigue during physical exertion, they often reduce their levels of physical activity. This inactivity exacerbates skeletal muscle atrophy and mitochondrial dysfunction, leading to further reductions in exercise capacity. Over time, the combination of central and peripheral dysfunction results in a progressive decline in overall functional capacity and quality of life, with patients becoming increasingly limited in their ability to perform even basic activities of daily living [33,34].

### 3.4. Exercise Training: Breaking the Vicious Cycle

**Figure 1:**
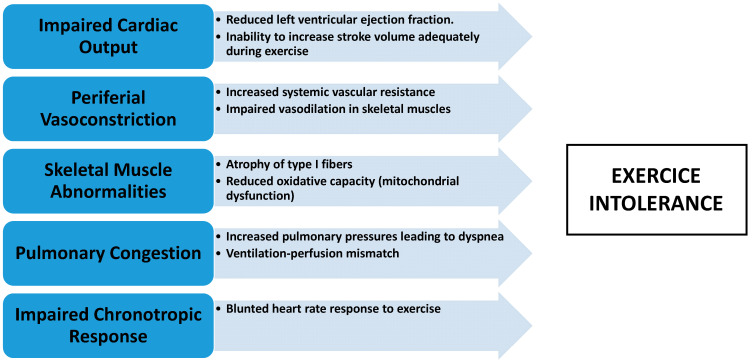
Mechanisms of exercise intolerance in HFrEF.

Despite the significant limitations imposed by HFrEF, exercise training has emerged as an effective intervention to break this cycle of inactivity and decline. Exercise training has been shown to improve both central hemodynamic function and peripheral muscle abnormalities, leading to improvements in exercise capacity, symptom relief, and quality of life in HFrEF patients [35]. By targeting both the central and peripheral mechanisms of exercise intolerance, exercise training can reverse many of the maladaptive changes associated with HFrEF, including improving cardiac output, enhancing endothelial function, increasing skeletal muscle oxidative capacity, and reducing inflammation [36]. This underscores the importance of incorporating structured exercise programs into the comprehensive management of HFrEF (Figure 1).

## 4. Physiological Modifications Induced by Exercise in HFrEF

Exercise training in patients with HFrEF leads to a series of beneficial physiological adaptations that improve cardiovascular function, skeletal muscle performance, and metabolic efficiency. These adaptations occur across multiple organ systems and contribute to improved exercise capacity, reduced symptoms, and better clinical outcomes. The beneficial effects of exercise in HFrEF are well documented, with structured programs demonstrating significant improvements in both morbidity and mortality.

### 4.1. Cardiac Output and Stroke Volume

One of the primary benefits of exercise training in HFrEF patients is the improvement in cardiac output and stroke volume, which are key determinants of exercise capacity. During exercise, the heart must increase its output to meet the heightened metabolic demands of the body. In HFrEF patients, this ability is severely compromised due to impaired systolic function. However, regular aerobic exercise has been shown to partially restore the heart’s capacity to increase stroke volume and cardiac output during physical activity [37].

The mechanism by which exercise improves cardiac output in HFrEF involves several factors, including enhanced left ventricular filling, reduced systemic vascular resistance, and improved contractility. Exercise promotes favorable cardiac remodeling by reducing left ventricular end-diastolic and end-systolic volumes, thereby improving systolic function [30]. This is supported by data from the HF-ACTION trial, which demonstrated that patients who underwent exercise training had significant increases in left ventricular ejection fraction (LVEF), reflecting better overall cardiac performance [38].

In addition to improving LVEF, exercise training enhances diastolic function, which is critical for maintaining an adequate stroke volume during physical activity [39]. Diastolic dysfunction, characterized by impaired ventricular relaxation and elevated filling pressures, is a common feature in HFrEF and contributes to exercise intolerance. Studies have shown that exercise training improves diastolic filling by enhancing the compliance of the left ventricle and reducing filling pressures [40]. These improvements in diastolic function translate into a better stroke volume and cardiac output during exercise, allowing patients to engage in physical activity with fewer symptoms of fatigue and shortness of breath.

### 4.2. Vascular Function and Endothelial Health

Exercise training has profound and well-documented effects on vascular function, particularly in improving endothelial health and reducing arterial stiffness, both of which are key factors in the pathophysiology of heart failure with reduced ejection fraction (HFrEF). Endothelial dysfunction, a common feature in HFrEF, is marked by the reduced bioavailability of nitric oxide (NO) and increased oxidative stress, both of which contribute significantly to exercise intolerance in these patients [41]. The endothelium, which lines blood vessels, plays a critical role in regulating vascular tone, and its dysfunction impairs the vasodilatory capacity of blood vessels, especially during physical exertion. This has direct implications for exercise capacity, as reduced blood flow to skeletal muscles limits oxygen delivery, contributing to early fatigue and reduced endurance.

#### 4.2.1. Role of Nitric Oxide in Vascular Function

Nitric oxide is a critical mediator of vasodilation and is essential for increasing blood flow to active muscles during exercise. It is produced by endothelial nitric oxide synthase (eNOS) in response to mechanical shear stress caused by increased blood flow during physical activity. However, in patients with HFrEF, endothelial cells exhibit impaired eNOS activity, leading to reduced NO production. This results in blunted vasodilation, inadequate oxygen delivery to muscles, and the early onset of fatigue during exercise. Additionally, the reduced availability of NO exacerbates vascular stiffness, increasing the workload on the failing heart by elevating systemic vascular resistance and afterload [42,43,44].

Exercise training has been shown to improve endothelial function by enhancing eNOS activity, thereby restoring NO production and increasing its bioavailability. This results in improved vasodilation, allowing for a better perfusion of skeletal muscles during exercise. Notably, exercise-induced improvements in NO-mediated vasodilation have been linked to enhanced exercise tolerance and a reduction in heart failure symptoms. Studies have demonstrated that regular aerobic exercise can significantly increase eNOS expression and activity in patients with HFrEF, facilitating better vascular responses during physical exertion [45].

#### 4.2.2. Oxidative Stress and Nitric Oxide Bioavailability

In HFrEF, oxidative stress plays a major role in the impairment of vascular function. The excessive production of reactive oxygen species (ROS), such as superoxide anions, contributes to the breakdown of NO, further diminishing its availability for vasodilation [46]. ROS production is elevated in heart failure due to chronic inflammation, neurohormonal activation, and mitochondrial dysfunction. This imbalance between NO production and ROS levels not only impairs endothelial function but also leads to vascular stiffness and contributes to the progression of heart failure.

Exercise training mitigates oxidative stress by enhancing the body’s antioxidant defenses and reducing the production of ROS. Aerobic exercise increases the expression of antioxidant enzymes, such as superoxide dismutase (SOD) and glutathione peroxidase, which neutralize ROS and prevent the degradation of NO. As a result, the preservation of NO bioavailability supports improved vasodilation, better oxygen delivery to muscles, and enhanced exercise performance in patients with HFrEF. These improvements in endothelial function and reduced oxidative stress are closely associated with increased exercise capacity and a reduction in heart failure symptoms, further highlighting the therapeutic benefits of exercise training in this population [47].

#### 4.2.3. Impact of Exercise on Arterial Stiffness

In addition to its effects on endothelial function, exercise training significantly reduces arterial stiffness, which is often elevated in patients with HFrEF. Arterial stiffness refers to the reduced elasticity of the arteries, primarily the large conduit arteries such as the aorta, and is a key determinant of increased afterload in heart failure. Elevated arterial stiffness increases the workload on the heart by raising systolic blood pressure and left ventricular wall stress, leading to worsening heart failure symptoms and the further deterioration of cardiac function. This increased afterload can also impair the heart’s ability to pump effectively, exacerbating the hemodynamic limitations observed in HFrEF [48].

Exercise training, particularly aerobic exercise, has been shown to reduce arterial stiffness by promoting favorable structural changes within the arterial wall [49]. These changes include increased elastin content, which enhances the elastic properties of the arteries, and reduced collagen deposition, which decreases arterial rigidity. By improving arterial compliance, exercise training reduces afterload, decreases myocardial oxygen demand, and alleviates the burden on the left ventricle, all of which contribute to improved cardiac function and symptom relief in HFrEF patients. Furthermore, these changes in arterial structure are accompanied by reductions in pulse wave velocity, a marker of arterial stiffness, further indicating the beneficial effects of exercise on vascular health [50].

#### 4.2.4. Exercise-Induced Improvements in Microvascular Function

In addition to its effects on large conduit arteries, exercise training also improves microvascular function, which plays a critical role in tissue perfusion during exercise. Microvascular dysfunction in HFrEF contributes to reduced capillary density and impaired blood flow distribution within skeletal muscles, further limiting exercise capacity. Exercise training enhances microvascular health by increasing capillary density and improving the function of small arterioles, which regulate blood flow at the tissue level [51]. These improvements in microvascular function help ensure more efficient oxygen delivery to exercising muscles, thereby enhancing aerobic capacity and reducing the sensation of fatigue during physical activity [52].

The enhanced microvascular function observed with exercise training is largely mediated by increased NO production and improved endothelial responsiveness in the microcirculation. Additionally, exercise promotes angiogenesis—the formation of new blood vessels—which further enhances tissue perfusion and oxygenation [53]. These microvascular adaptations are particularly important in patients with HFrEF as they help counteract the peripheral limitations to exercise capacity imposed by skeletal muscle abnormalities and endothelial dysfunction.

#### 4.2.5. Clinical Relevance of Vascular Improvements in HFrEF

The vascular benefits of exercise training in patients with heart failure with reduced ejection fraction (HFrEF) extend far beyond simply improving exercise tolerance. Enhanced endothelial function, reduced oxidative stress, and decreased arterial stiffness—key vascular improvements brought about by regular exercise—have profound and direct implications for clinical outcomes in this population. Numerous studies have demonstrated that exercise-induced vascular improvements are associated with significant reductions in hospitalizations, improved functional status, and, most importantly, lower mortality rates in HFrEF patients [54]. This is particularly important given that heart failure remains a leading cause of morbidity and mortality worldwide, with frequent hospital readmissions contributing to the disease’s burden on healthcare systems.

##### Reduced Hospitalizations and Morbidity

Improved vascular health resulting from exercise training leads to a better regulation of blood pressure, enhanced cardiac output, and more efficient oxygen delivery to peripheral tissues. These changes help alleviate the hemodynamic stress placed on the heart, reducing the frequency and severity of acute decompensations of heart failure that often require hospitalization. By improving endothelial function, increasing nitric oxide (NO) availability, and reducing oxidative stress, exercise effectively reduces the burden on both the central cardiovascular system and peripheral vascular networks. As a result, HFrEF patients experience fewer exacerbations of their symptoms, and the need for acute interventions, including diuretics and hospital-based treatments, diminishes.

The ability of exercise to modulate arterial stiffness also plays a crucial role in reducing hospitalizations. Arterial stiffness, commonly seen in HFrEF patients, exacerbates afterload on the heart, leading to the worsening of heart failure symptoms. By improving arterial elasticity, exercise lowers systemic vascular resistance, reduces afterload, and contributes to a more stable hemodynamic profile, further preventing acute decompensations that would necessitate hospitalization. Studies have shown that patients with HFrEF who engage in regular exercise experience not only improved physical capacity but also a substantial decrease in the number of heart failure-related hospitalizations [55,56,57].

##### Slowing Adverse Cardiac Remodeling

One of the most significant clinical benefits of improved vascular function through exercise training is its role in mitigating adverse cardiac remodeling—a hallmark of HFrEF. The chronic activation of compensatory neurohormonal pathways, including the renin–angiotensin–aldosterone system (RAAS) and the sympathetic nervous system (SNS), leads to progressive structural changes in the heart, such as left ventricular dilation and fibrosis. These changes further impair cardiac function, worsen heart failure, and are associated with poor long-term outcomes.

Exercise training, by reducing oxidative stress and enhancing NO bioavailability, has been shown to attenuate the pathological processes that drive adverse cardiac remodeling. Improved endothelial function and reduced arterial stiffness lower the heart’s workload, allowing the left ventricle to function more efficiently and preventing the progressive dilation and fibrosis that characterize HFrEF. Additionally, exercise-induced improvements in vascular health contribute to better coronary perfusion, which is crucial for maintaining myocardial oxygen supply and preventing ischemia-induced myocardial damage [58,59].

##### Improved Symptom Management and Quality of Life

The vascular improvements associated with exercise training also have a direct impact on symptom management and the overall quality of life for patients with HFrEF. Patients often experience debilitating symptoms, including dyspnea, fatigue, and exercise intolerance, all of which severely limit their daily activities and overall well-being [60]. Exercise-induced improvements in endothelial function and vascular health allow for more effective blood flow distribution to working muscles during physical activity, reducing the sensation of fatigue and improving exercise tolerance. Moreover, the reduction in oxidative stress and arterial stiffness helps to relieve breathlessness and other symptoms related to elevated afterload and poor peripheral perfusion [61,62].

Enhanced vascular function also contributes to greater psychological well-being and social engagement. Patients with HFrEF who participate in regular exercise often report significant improvements in their mental health and quality of life, likely as a result of their increased physical capabilities and reduced symptom burden [63]. The psychological benefits of exercise are important, as depression and anxiety are common in heart failure patients, often compounding the disease’s physical symptoms and leading to worse outcomes. By improving both physical and vascular health, exercise training thus addresses the broader spectrum of quality of life issues faced by HFrEF patients.

##### Long-Term Prognosis and Mortality

Perhaps the most important clinical relevance of exercise-induced vascular improvements lies in their impact on long-term prognosis and survival. Enhanced endothelial function, reduced arterial stiffness, and improved microvascular function all contribute to a more stable cardiovascular profile, which is linked to a reduction in the progression of heart failure and an overall improvement in long-term outcomes [64]. Research has shown that patients with HFrEF who engage in structured exercise programs not only experience fewer cardiovascular events but also demonstrate higher survival rates compared to their sedentary counterparts [65].

The reduction in mortality associated with exercise training is likely multifactorial, involving improvements in vascular health, cardiac function, and peripheral muscle efficiency. By addressing the central and peripheral contributors to heart failure progression, including oxidative stress, endothelial dysfunction, and arterial stiffness, exercise reduces the likelihood of fatal cardiovascular events such as sudden cardiac death, which is a major cause of mortality in HFrEF patients [66]. Additionally, exercise promotes better autonomic balance by reducing sympathetic overactivity and increasing parasympathetic tone, both of which are associated with improved survival in heart failure [67].

##### Exercise as an Adjunct to Pharmacotherapy

While pharmacological therapies remain the cornerstone of HFrEF management, exercise training provides a powerful adjunct to these treatments, offering additional benefits that medications alone may not achieve. Angiotensin-converting enzyme inhibitors (ACE inhibitors), beta-blockers, and mineralocorticoid receptor antagonists all target neurohormonal pathways to slow disease progression, but they may not fully address the peripheral vascular and muscular abnormalities seen in HFrEF. Exercise, by improving endothelial function, reducing arterial stiffness, and enhancing microvascular health, complements the effects of these medications, leading to a more comprehensive management of heart failure symptoms and progression.

Furthermore, regular exercise has been shown to enhance the efficacy of pharmacological treatments by improving drug uptake and responsiveness. Patients who maintain an active lifestyle often experience better blood pressure control, more stable heart rates, and greater improvements in left ventricular function, all of which contribute to the overall success of medical management in HFrEF. In this regard, exercise serves as a critical non-pharmacological intervention that not only improves vascular health but also optimizes the therapeutic effects of heart failure medications.

High-intensity training (HIT) has emerged as a promising intervention for patients with heart failure, offering unique benefits compared to traditional exercise modalities. HIT involves short bursts of intense activity followed by rest or low-intensity periods, significantly improving cardiovascular fitness and functional capacity in heart failure patients. Research shows that this training approach can lead to greater improvements in peak oxygen consumption and exercise tolerance than moderate-intensity continuous training (MICT).

The physiological basis for these benefits lies in HIT’s ability to induce greater adaptations in both skeletal muscle and cardiovascular systems. The brief, intense efforts push the body to its anaerobic threshold, enhancing mitochondrial density and oxidative capacity within muscle fibers. This adaptation not only improves muscle efficiency but also contributes to better glucose and lipid metabolism, which are often impaired in heart failure patients.

In addition to HIT, other training modalities, such as resistance training and aerobic exercise, also play important roles in heart failure management. Resistance training focuses on building muscle strength, which is crucial for maintaining physical function and improving quality of life. This type of training helps counteract skeletal muscle dysfunction—a common consequence of heart failure—by promoting muscle hypertrophy and increasing muscle mass.

Aerobic exercise, typically performed at a moderate intensity, remains a foundational component of rehabilitation programs. It helps improve cardiovascular health by enhancing cardiac output and reducing resting heart rate, ultimately leading to better exercise capacity and reduced symptoms of fatigue.

While HIT shows considerable promise for enhancing functional capacity, the best approach often combines various modalities tailored to individual patient needs. Incorporating HIT, resistance training, and aerobic exercise can maximize benefits, improve adherence, and address the multifaceted challenges of heart failure, leading to improved overall health and well-being for patients.

SGLT2 inhibitors (SGLT2is) have rapidly gained recognition as a transformative class of medications in the management of heart failure, particularly when combined with exercise. These medications work primarily by preventing the reabsorption of glucose in the kidneys, which leads to increased glucose excretion and diuresis. This mechanism not only helps control blood sugar levels in patients with diabetes but also alleviates fluid overload in heart failure patients, improving symptoms such as dyspnea and edema.

When SGLT2is are integrated with an exercise regimen, the benefits become even more pronounced. Regular physical activity enhances cardiovascular fitness and muscle strength, addressing the common issues of deconditioning and skeletal muscle dysfunction associated with heart failure. Exercise can improve insulin sensitivity, enhance metabolic flexibility, and promote weight loss, further complementing the effects of SGLT2is.

Recent studies have highlighted the synergistic effects of combining SGLT2is with exercise, showing that this approach leads to improved functional capacity, reduced hospitalizations, and a better quality of life for patients. The dual impact of SGLT2is on both metabolic and cardiovascular parameters creates a comprehensive strategy to combat the multifaceted challenges of heart failure.

Furthermore, the combination of SGLT2is and exercise has been linked to favorable changes in cardiac remodeling and reduced inflammation, providing additional protection against heart failure progression. This integrated approach not only addresses immediate symptoms but also targets underlying pathophysiological mechanisms, offering a holistic treatment paradigm.

As healthcare providers increasingly recognize the importance of lifestyle interventions, the combination of SGLT2 inhibitors and exercise emerges as a promising strategy for enhancing patient outcomes in heart failure management. By promoting adherence to both pharmacological and non-pharmacological treatments, clinicians can empower patients to take an active role in their health, leading to improved long-term results [68,69].

##### Public Health and Healthcare Implications

The vascular improvements brought about by exercise training in HFrEF also have significant implications for public health and healthcare systems. Given the rising prevalence of heart failure and the associated healthcare costs, incorporating exercise-based interventions into standard heart failure care has the potential to reduce the economic burden of the disease. By decreasing hospitalizations, lowering the need for advanced heart failure therapies, and improving overall cardiovascular health, exercise training offers a cost-effective strategy for improving outcomes in this growing patient population [70].

### 4.3. Skeletal Muscle Adaptations

Skeletal muscle dysfunction plays a central role in the exercise intolerance observed in HFrEF patients. As mentioned earlier, patients with HFrEF often exhibit reduced capillary density, mitochondrial dysfunction, and a shift in muscle fiber composition towards more glycolytic fibers. These abnormalities contribute to early muscle fatigue and reduced endurance during exercise [71].

Exercise training reverses many of these skeletal muscle abnormalities by promoting capillary growth (angiogenesis), enhancing mitochondrial biogenesis, and restoring a more favorable balance of oxidative and glycolytic muscle fibers. Angiogenesis improves oxygen delivery to muscles during exercise, while increased mitochondrial content enhances the capacity for aerobic energy production. These adaptations result in greater muscle efficiency and a delayed onset of fatigue, allowing patients to exercise for longer periods with less effort [72].

Additionally, exercise training increases muscle mass and strength by stimulating anabolic pathways that promote muscle protein synthesis. Resistance training, in particular, has been shown to increase the muscle cross-sectional area and improve overall muscle function in HFrEF patients. Improved muscle strength reduces the oxygen cost of physical activity, making daily tasks such as walking or climbing stairs less strenuous. As a result, patients experience a greater ability to perform activities of daily living and an overall improvement in quality of life.

### 4.4. Biochemical and Analytical Modifications

Exercise training has significant effects on various biochemical and analytical parameters in patients with HFrEF, contributing to metabolic improvements and reductions in systemic inflammation. One of the primary biochemical adaptations observed with exercise is the reduction in inflammatory markers, including C-reactive protein (CRP) and tumor necrosis factor-alpha (TNF-α). Chronic inflammation is a known contributor to the progression of heart failure and is associated with poor clinical outcomes. Regular exercise helps attenuate this inflammatory response, leading to improved outcomes in HFrEF patients.

In addition to reducing inflammation, exercise positively influences lipid profiles by lowering low-density lipoprotein (LDL) cholesterol and triglyceride levels while increasing high-density lipoprotein (HDL) cholesterol. These changes reduce the risk of atherosclerosis and cardiovascular events, which are particularly important in HFrEF patients who are at an increased risk of coronary artery disease [73,74].

Furthermore, exercise has favorable effects on glucose metabolism and insulin sensitivity. HFrEF patients often exhibit insulin resistance, which can exacerbate heart failure symptoms. Regular physical activity improves glucose uptake in skeletal muscles and enhances insulin sensitivity, thereby reducing the risk of diabetes and improving overall metabolic health [75].

Another important biochemical parameter affected by exercise is renal function. HFrEF patients frequently experience renal impairment due to reduced cardiac output and renal perfusion. Exercise training has been associated with improvements in renal function, likely mediated by enhanced cardiac output, reduced inflammation, and improved hemodynamics. These improvements can help mitigate the progression of chronic kidney disease in this population [76,77].

**Figure 2:**
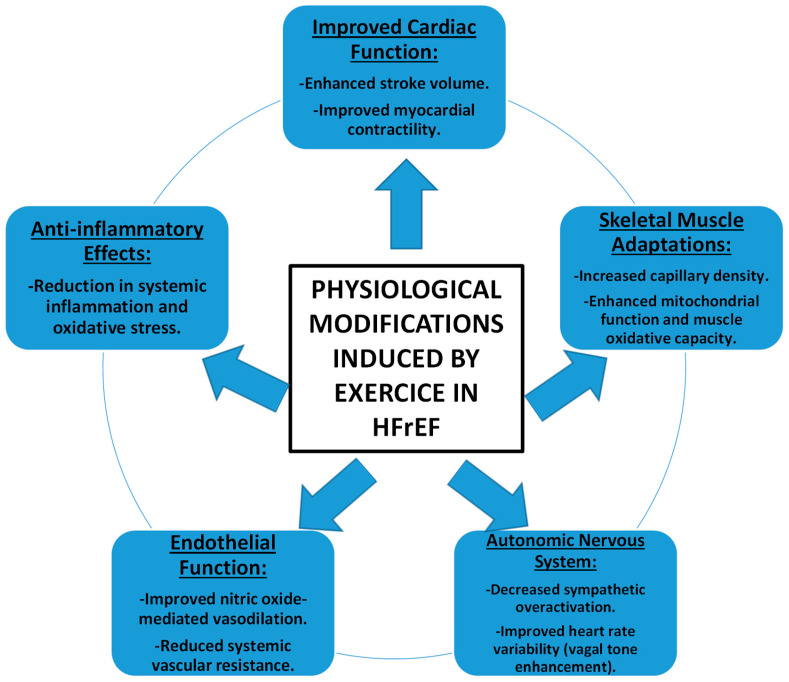
Physiological modifications induced by exercise in HFrEF.

Lastly, exercise training can lead to changes in biomarkers associated with heart failure severity, including brain natriuretic peptide (BNP) levels. Reduced BNP levels following exercise interventions indicate improved cardiac function and reduced heart failure severity, contributing to better clinical outcomes [78] (Figure 2).

## 5. Conclusions and Future Directions

Exercise is a potent intervention for modifying numerous physiological, biochemical, and analytical parameters in patients with HFrEF. Structured exercise programs lead to improvements in cardiac function, vascular health, muscle performance, and metabolic control. These changes contribute to better clinical outcomes, including improved functional capacity, reduced symptoms, and lower mortality rates [79].

Future research should focus on personalizing exercise interventions for individual patients based on their baseline characteristics, comorbidities, and genetic factors. Identifying the most effective types, intensities, and durations of exercise training that yield the greatest benefits in HFrEF patients is crucial. Large-scale clinical trials are also needed to determine the optimal exercise modalities and intensities that yield the greatest long-term benefits for HFrEF patients [80].

Furthermore, studies investigating the molecular and cellular mechanisms underlying the benefits of exercise in HFrEF will enhance our understanding of how exercise mediates these effects. This knowledge will aid in the development of targeted exercise prescriptions and rehabilitation programs, ensuring that patients with HFrEF receive the most effective and individualized care [81,82].

## Data Availability

No new data were created or analyzed in this study.
